# Skin-Derived ABCB5^+^ Mesenchymal Stem Cells for High-Medical-Need Inflammatory Diseases: From Discovery to Entering Clinical Routine

**DOI:** 10.3390/ijms24010066

**Published:** 2022-12-21

**Authors:** Elke Niebergall-Roth, Natasha Y. Frank, Christoph Ganss, Markus H. Frank, Mark A. Kluth

**Affiliations:** 1 TICEBA GmbH, 69120 Heidelberg, Germany; 2 Department of Medicine, VA Boston Healthcare System, Boston, MA 02132, USA; 3 Division of Genetics, Brigham and Women’s Hospital, Harvard Medical School, Boston, MA 02115, USA; 4 Harvard Stem Cell Institute, Harvard University, Cambridge, MA 02138, USA; 5 Transplant Research Program, Boston Children’s Hospital, Harvard Medical School, Boston, MA 02115, USA; 6 RHEACELL GmbH & Co. KG, 69120 Heidelberg, Germany; 7 Department of Dermatology, Brigham and Women’s Hospital, Harvard Medical School, Boston, MA 02115, USA; 8 School of Medical and Health Sciences, Edith Cowan University, Perth 6027, Australia

**Keywords:** ABCB5, advanced-therapy medicinal product, angiogenesis, cell therapy, chronic wound, epidermolysis bullosa, immunomodulation, inflammation, mesenchymal stem cells, wound healing

## Abstract

The ATP-binding cassette superfamily member ABCB5 identifies a subset of skin-resident mesenchymal stem cells (MSCs) that exhibit potent immunomodulatory and wound healing-promoting capacities along with superior homing ability. The ABCB5^+^ MSCs can be easily accessed from discarded skin samples, expanded, and delivered as a highly homogenous medicinal product with standardized potency. A range of preclinical studies has suggested therapeutic efficacy of ABCB5^+^ MSCs in a variety of currently uncurable skin and non-skin inflammatory diseases, which has been substantiated thus far by distinct clinical trials in chronic skin wounds or recessive dystrophic epidermolysis bullosa. Therefore, skin-derived ABCB5^+^ MSCs have the potential to provide a breakthrough at the forefront of MSC-based therapies striving to fulfill current unmet medical needs. The most recent milestones in this regard are the approval of a phase III pivotal trial of ABCB5^+^ MSCs for treatment of recessive dystrophic and junctional epidermolysis bullosa by the US Food and Drug Administration, and national market access of ABCB5^+^ MSCs (AMESANAR^®^) for therapy-refractory chronic venous ulcers under the national hospital exemption pathway in Germany.

## 1. Introduction

Despite enormous progress in medical research and development, there are still various chronic or debilitating diseases that cannot be treated satisfactorily. The multifaceted biological characteristics of mesenchymal stem cells (MSCs) in concert with their ability to sense and adaptively respond to their environment [1] have made these cells promising candidates for treatment of a broad range of currently uncurable conditions. However, while numerous phase I/II clinical trials have suggested therapeutic efficacy, only very few late-stage trials have confirmed the expected clinical benefit [2,3], with significant proportions of patients failing to respond to therapy [4]. As a result, more than 25 years since the first published clinical trial of ex vivo-expanded MSCs in 1995 [5], fewer than ten MSC-based cell therapy products have gained marketing authorization at least on a national level [6] (Table 1).

**Figure 1:**
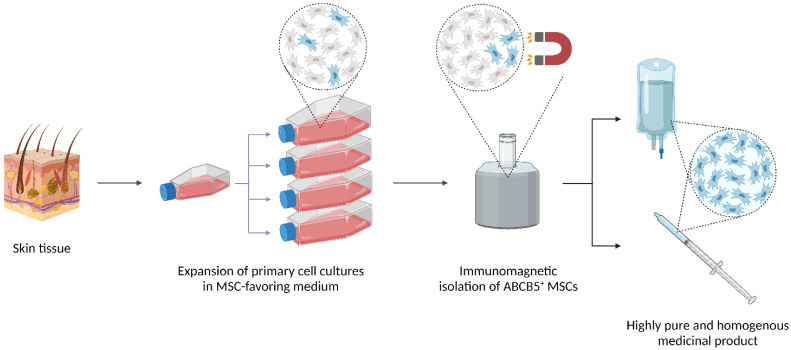
Schematic representation of the good practice-compliant manufacturing process of ABCB5^+^ mesenchymal stem cells (MSCs). Human skin tissue is enzymatically digested, and cells are expanded as unsegregated culture upon adherence selection in an in-house MSC-favoring medium. After serial passaging for up to 16 passages, ABCB5^+^ cells are isolated by immunomagnetic bead cell sorting using an antibody directed against an extracellular sequence of the ABCB5 molecule. The final medicinal product is formulated by thawing and reconstituting isolated, cryopreserved ABCB5^+^ MSCs in Ringer’s lactated solution supplemented with human serum albumin and glucose in infusion bags or syringes as needed. While initially all cells were supplied as reconstituted ready-to-use products, they can now also be delivered as frozen vials on dry ice to be thawed and reconstituted at the clinical site, which provides for prolonged transport stability required for worldwide shipping. Created with BioRender.com.

Among several factors, the remarkable plasticity and heterogeneity of MSCs over time and when exposed to different microenvironments has been claimed to significantly contribute to suboptimal and inconsistent clinical outcomes of MSC-based treatment approaches, pointing to the need for identification of appropriate cellular markers that would provide functional discrimination [7,8]. During the past years, the plasma membrane-spanning P-glycoprotein ATP-binding cassette subfamily B member 5 (ABCB5) [9] has emerged as a feasible surface marker identifying a novel dermal subpopulation of MSCs that exhibit distinct immunomodulatory properties [10,11]. Using a specific antibody directed against an extracellular-loop sequence of the ABCB5 molecule [9], ABCB5^+^ MSCs can be reliably isolated from expansion cultures to define highly pure and homogenous cell populations that fulfil the standards of good manufacturing practice (GMP) [12] (Figure 1). A further major advantage of ABCB5^+^ MSCs over classically used MSCs derived from bone marrow (BM-MSCs) or adipose tissue (AT-MSCs) is that ABCB5^+^ MSCs can be easily obtained from discarded skin tissues donated by people undergoing plastic surgeries, without the need for ad-hoc invasive procedures [12].

Together, ABCB5^+^ MSCs may have the potential to achieve a breakthrough at the forefront of MSC-based therapies, making a significant contribution toward fulfilling current unmet medical needs. Here, we delineate the research conducted so far to characterize ABCB5^+^ MSCs in situ and following ex vivo expansion, to uncover their physiological and biopharmacological properties, and to explore potential indications for ABCB5^+^ MSCs manufactured as an GMP-conforming advanced-therapy medicinal product (ATMP).

## 2. ABCB5 in Physiological and Cancer Stem Cells

The efflux transporter ABCB5 was detected as the third member of the P-glycoprotein family of ATP-binding cassette multidrug resistance (MDR) transporters [13] next to its structural paralogs ABCB1 (MDR1, CD243) [14,15] and ABCB4 (MDR3) [16]. Originally, ABCB5 was characterized on CD133^+^ human epidermal melanocyte progenitor cells, acting as a negative regulator of cell differentiation, and on human malignant melanoma cells, serving as a chemoresistance-conferring drug efflux transporter [9]. Following its initial discovery, ABCB5 has been shown to be highly expressed by cancer stem cells in various human malignancies including malignant melanoma [9,17,18,19,20], colorectal cancer [21,22], hepatocellular carcinoma [23], breast cancer [24], non-small cell lung cancer [25], oral squamous cell carcinoma [26], glioblastoma [27], and Merkel cell carcinoma [28], where it is associated with tumor growth, invasiveness, chemoresistance and recurrence [20,29,30].

In normal tissues, the presence of ABCB5 in cell types with secretory and excretory functions in the gastrointestinal tract, liver, pancreas, kidney and at the blood-brain and blood-testis barriers has suggested a physiological function of ABCB5 in cellular extrusion of deleterious xenobiotics and endogenous metabolites [31]. In the human placenta, ABCB5 is expressed in the cytotrophoblast layer of placental villi, where it might be involved in fetal protection from xenobiotic and immunogenic factors [32]. In the mouse and human eye, ABCB5 mediates normal stem cell maintenance via antiapoptotic pathways [33] in mouse and human limbal stem cells that are required to maintain corneal development, homeostasis, and repair [33,34,35], and it is also expressed on mouse retinal pigment epithelium progenitor cells involved in retinal development and regeneration [33,36] and epithelial progenitor cells in human lacrimal gland tissue [37]. In mouse and human skin, ABCB5 was found to identify a population of dermal immunomodulatory cells [10] with a surface marker expression similar to conventional mesenchymal stromal cells and functional properties distinct from dermal fibroblasts [10,11].

## 3. Biological Properties of ABCB5^+^ MSCs

Immunostaining of healthy human skin sections revealed dermal ABCB5^+^ cell subsets to reside in the reticular dermis at frequencies ranging from 1.5% to 4.0% of dermal cells [10,11]. These cells are frequently confined to a perivascular endogenous niche in close association with CD31^+^ endothelial cells or found dispersed within the interfollicular dermis independent of hair follicles [11].

The ABCB5^+^ cells derived from enzymatically digested skin and separated by multiple rounds of magnetic-bead cell sorting using an anti-ABCB5 monoclonal antibody [9] are plastic-adherent, display a spindle-like, fibroblastoid cell morphology, exhibit a typical mesenchymal-lineage marker expression pattern [10,11] (summarized in Table 2), and demonstrate a consistent and significantly increased potential for in vitro adipogenic, osteogenic, and chondrogenic lineage differentiation as compared with donor-matched ABCB5^–^ dermal fibroblasts [11]. In another contrast to ABCB5^−^ fractions, ABCB5^+^ dermal cells were shown to give rise to single cell-derived colonies, with clonal colonies generated from single cells again displaying clonogenic growth. Remarkably, 76% of these self-renewed clones maintained their trilineage differentiation potential, and all self-renewed clones could differentiate into at least one mesenchymal lineage [11]. In addition, studies in murine muscle injury and skin wound models have indicated in vivo myogenic and endothelial differentiation potential, respectively [38,39] (see Section 5.1. (Trans-)Differentiation).

Together, dermal ABCB5^+^ cells display morphological and functional properties similar to conventional mesenchymal stromal cells [40] and, in addition, possess self-renewal and differentiation capacity in vitro and in vivo, thereby meeting the criteria for classification as a mesenchymal stem cell (MSC) [41] and substantiating the conclusion that ABCB5^+^ identifies a MSC population amongst dermal mesenchymal stromal cells.

## 4. Physiological Functions of ABCB5^+^ MSCs

### 4.1. Stem Cell Integrity and Quiescence

The expression of ABC transporters including ABCB5 by stem cells has predominantly been ascribed to their ability of preventing toxic compounds from entering the cell and effluxing secondary metabolites out of the cell. This is thought to confer protection to these long-lived cell subsets against xenobiotics, thereby contributing to maintenance of stem cell integrity [31,42].

In addition to these protective functions, ABCB5 expressed on dermal MSCs appears to be involved in cell cycle regulation. Specifically, blocking ABCB5 function on ABCB5^+^ MSCs in vitro, using a neutralizing anti-ABCB5 antibody, resulted in an increase in actively proliferating ABCB5^+^ MSCs [43]. This finding supports a role for ABCB5 as a mediator of stem cell quiescence of ABCB5^+^ MSCs, as was previously demonstrated for other ABCB5-expressing stem cell types such as limbal stem cells and cancer stem cells [20,33].

**Table 2:** Cell marker expression pattern of ABCB5^+^ MSCs.

Cell Marker	Typically Expressed by	Expression by ABCB5^+^ MSCs	Detection Method	References
CD73	MSCs	Yes	FCM	[10,11]
CD90	MSCs	Yes	FCM	[11]
CD105	MSCs	Yes	FCM	[10,11]
CD29	MSCs	Yes	FCM	[10]
CD44	MSCs	Yes	FCM	[10]
CD49e	MSCs	Yes	FCM	[10]
CD166	MSCs	Yes	FCM	[10]
CD14	Monocytes/macrophages	No	FCM	[11]
CD20	B lymphocytes	No	FCM	[11]
CD34	Hematopoietic-lineage cells, dendritic cells	No	IF	[10]
			FCM	[10,11]
CD45	Hematopoietic-lineage cells	No	FCM	[10,11]
CD31	Endothelial-lineage cells	No	IF	[10,11]
			FCM	[10]
NG2	Pericytes	No	IF	[11]
CD318	Epithelial cells	No	FCM	[11]
MelanA	Melanocytic cells	No	IF	[44]
			FCM	[11]
CD133	Cancer stem cells, malignant melanoma cells	No	IF	[44]
			FCM	[11]
LGR5	Hair follicle stem cells	No	IF	[44]
LNGFR/CD271	Neuro-ectodermal skin-derived precursors	No	FCM	[11]
SSEA-4	Stem cells	Yes	IF, FCM	[11]
SOX2	Stem cells	Yes	IF	[11]
POU5F1/Oct4	Stem cells	Yes	IF	[11]
DPP-4/CD26	Upper-lineage fibroblasts	Yes	IF	[11]
PRDM1/BLIMP-1	Upper-lineage fibroblasts	Yes	IF	[11]
α-SMA	Lower-lineage fibroblasts	No	IF	[11]

BLIMP-1—B lymphocyte-induced maturation protein 1; CD—cluster of differentiation; DPP-4—dipeptidyl-peptidase 4; FCM—flow cytometry; IF immunofluorescence staining; LGR5—leucine-rich G protein-coupled receptor 5; LNGFR—low-affinity nerve growth factor receptor; NG2—neural/glial antigen 2; Oct4—octamer-binding transcription factor 4; POU5F1—POU domain class 5 transcription factor 1; PRDM1—PR domain zinc finger protein 1; α-SMA—α-smooth muscle actin; SOX2—Sex-determining region Y box 2; SSEA—stage-specific embryonic antigen 4.

### 4.2. Cutaneous Regeneration and Wound Healing

Indications for a role of ABCB5^+^ MSCs in normal skin regeneration have come from age-related changes in numbers, niche preferences and differentiation capacities of dermal ABCB5^+^ MSCs, which correlate with the reduced regenerative capacity of the aging skin. Specifically, immunostaining analyses of a series of human skin specimens revealed a significant decline of dermal ABCB5^+^ MSC frequency from about 3.2% of all dermal cells in young individuals (≤20 years) to about 1.6% in individuals aged above 70 years [44]. At the level of gene expression, age-dependent increases in the expression of the DNA double-strand break marker γH2AX, of various genes involved in the nucleotide excision repair pathway and of pro-apoptotic genes including p73 suggest that accumulation of DNA damage and enhanced apoptosis represent significant challenges to the integrity and numbers of ABCB5^+^ MSCs in old individuals [45].

The age-dependent decline in ABCB5^+^ MSCs was shown to be concomitant with a change in niche preference of ABCB5^+^ MSCs from a predominant (75%) perivascular localization in close association to endothelial cells of small vessels in the skin of young individuals to a predominant (90%) interfollicular localization in the skin of old individuals [44]. Both the decrease in number and the perivascular niche preference of ABCB5^+^ MSCs coincided with a decrease of perivascular osteopontin, an extracellular matrix (ECM) component provided by perivascular neural glial antigen 2-expressing niche pericytes [46]. Interestingly, in an osteopontin-depleted mouse model, numbers of dermal ABCB5^+^ MSCs were even lower than in aged wild-type mice, pointing to a pivotal role of osteopontin in the regulation of dermal ABCB5^+^ MSC biology [46]. Furthermore, ABCB5^+^ MSCs of older individuals showed a gradual decrease in the percentages of cells expressing the stem cell markers stage-specific embryonic antigen 4 and sex-determining region Y box 2 [46], along with an enhanced adipogenic and markedly reduced osteogenic and chondrogenic differentiation potential as compared with ABCB5^+^ MSCs of young individuals [45,46].

Collectively, a robust decrease in ABCB5^+^ MSCs together with distinct changes in niche preference and differentiation capacity may contribute to the reduced regenerative capacity of the aged skin, in turn suggesting critical roles for ABCB5^+^ MSCs in normal skin homeostasis and physiological regeneration [43,44,45]. Moreover, ABCB5 has been found to be functionally involved in cutaneous wound healing through molecular regulation of a proangiogenic pAkt/hypoxia-inducible factor (HIF)-1α/vascular endothelial growth factor (VEGF) cascade, based on delayed wound closure, increased inflammatory stroma thickness and aberrant angiogenesis associated with impaired pAkt/HIF-1α/VEGF signaling observed in *Abcb5*-knockout as compared with wildtype mice [47].

## 5. Biopharmacological Modes of Action

Following the original discovery of MSCs in bone marrow more than 50 years ago [48,49], MSCs from various tissues have been extensively studied regarding their potential therapeutic capabilities. In response to signals associated with tissue injury, MSCs can promote tissue regeneration in different ways. While initial efforts focused on the MSCs’ multi-lineage differentiation potential, which suggested that MSCs could repair injured tissues through replacement of damaged cells [50,51,52,53,54,55], a growing body of evidence has shown that MSCs additionally possess a wide range of immunomodulatory, trophic and anti-microbial capacities, which act together to restore homeostasis and facilitate regenerative responses in inflamed, injured, diseased or infected tissues [1,56,57,58,59]. Therapeutic effects of MSCs can occur through direct cell-cell interactions and through paracrine routes [59,60,61,62]. Comparative studies have demonstrated that the functional properties of MSCs vary depending on their physiological niche [63,64,65,66], and it is now understood that MSCs comprise multiple subpopulations expressing specific surface markers, which may present various biological functions [67]. Therefore, any attempts to apply MSCs in a therapeutic context presuppose a thorough exploration of the mechanisms of action of the particular MSC subset under investigation.

### 5.1. (Trans-)Differentiation

**Figure 2:**
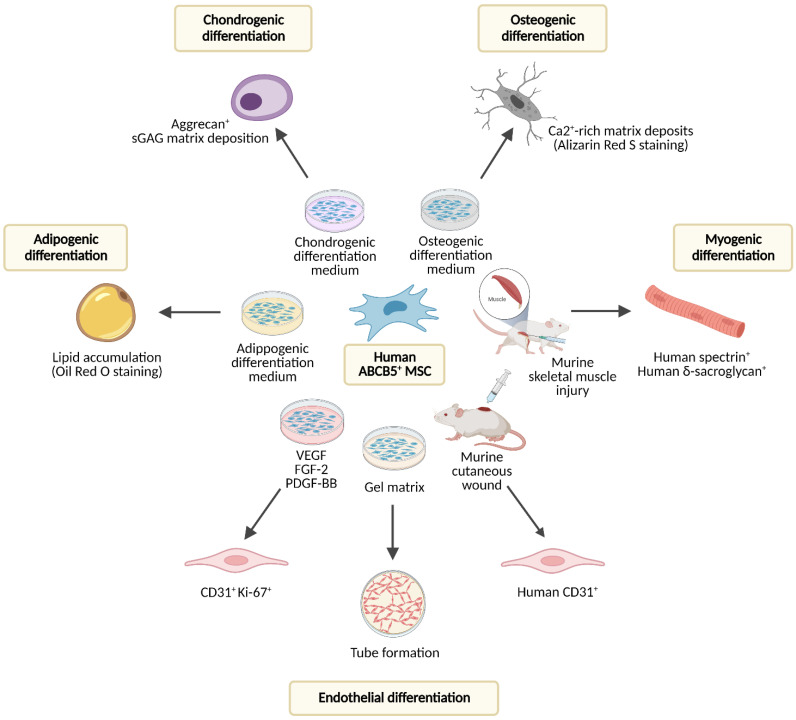
Differentiation potential of human dermal ABCB5^+^ MSCs studied in vitro and upon therapeutic application in mouse injury models. FGF-2—fibroblast growth factor 2; PDGF-BB—platelet-derived growth factor BB; VEGF—vascular endothelial growth factor. Created with BioRender.com.

As outlined above (see Section 3. Biological Properties of ABCB5^+^ MSCs), skin-derived ABCB5^+^ MSCs display the classic trilineage differentiation potential characteristic for multipotent mesenchymal stromal cells [40], i.e., they can differentiate into adipogenic, osteogenic and chondrogenic mesenchymal-lineage cells in vitro [11]. In addition, ABCB5^+^ MSCs are capable of adopting phenotypic and functional characteristics of actively proliferating endothelial cells, which was demonstrated by co-expression of CD31 and Ki-67 on ABCB5^+^ MSCs cultured in growth factor-supplemented medium and by formation of capillary-like structures comparable to human umbilical endothelial vein cells when ABCB5^+^ MSCs were seeded on gel matrix [68] (Figure 2).

The capacity of dermal ABCB5^+^ cells to differentiate into endothelial-lineage cells in vivo was supported by a preclinical study, in which topical application of human ABCB5^+^ MSCs, which in their undifferentiated state do not express CD31 [10,11], onto cutaneous wounds of NSG mice induced expression of human-specific CD31 in the wound tissue [39]. Moreover, in a mouse skeletal muscle injury model, human ABCB5^+^ MSCs gave rise to human spectrin- and δ-sarcoglycan-expressing skeletal myofibers while accelerating skeletal muscle regeneration, indicating myogenic differentiation potential [38] (Figure 2).

### 5.2. Immunomodulation

**Figure 3:**
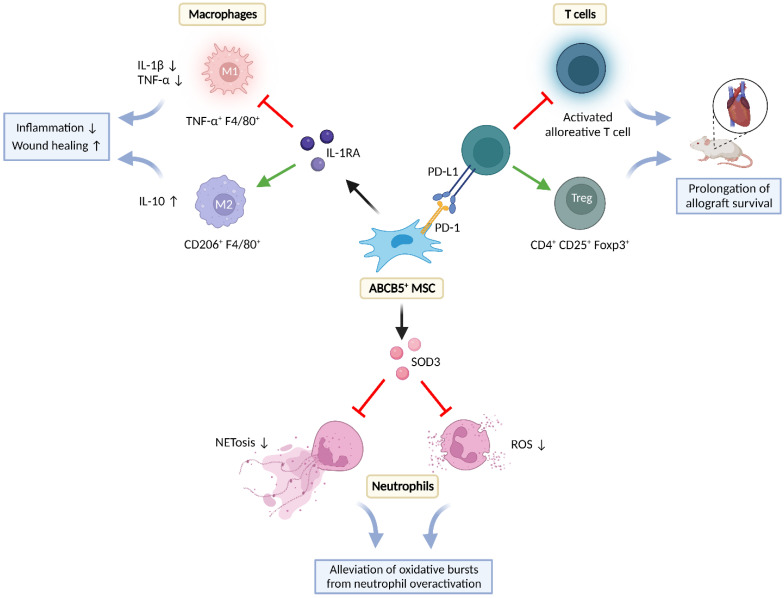
Immunomodulatory interactions of ABCB5^+^ MSCs with T-cells, macrophages, and neutrophils in inflamed environments. Foxp3—forkhead box protein P3; IL—interleukin; IL-1RA—interleukin 1 receptor antagonist; NET—neutrophil extracellular trap; PD-1—programmed cell death protein 1; PD-L1 —programmed cell death ligand 1; ROS—reactive oxygen species; SOD3—superoxide dismutase 3; TNF-α—tumor necrosis factor α. Created with BioRender.com.

Dermal ABCB5^+^ MSCs can modulate immune responses by interacting with cells of the innate (neutrophils, macrophages) and the adaptative immune system (T-cells) (Figure 3). Interaction can occur via direct, cell contact-dependent signaling and via paracrine secretion of regulatory molecules.

#### 5.2.1. Effects on T-Cells

The physiological niche of ABCB5^+^ MSCs, i.e., the mammalian dermis, contains large populations of effector T-cells [69,70,71]. This implicates that dermal immunity must be tightly controlled to prevent excessive activation, while maintaining the ability to efficiently fight invading noxious pathogens [72]. In this regard, the immune checkpoint receptor programmed cell death protein 1 (PD-1) expressed by activated T-cells, B-cells and myeloid cells plays an essential role in regulating T-cell immunity [73,74,75]. Intriguingly, ABCB5^+^ MSCs, unlike other mesenchymal cell types in the human and murine dermis, also co-express PD-1. The demonstration of PD-1 expression on a skin-specific, non-lymphocytic (CD45^−^) cell population suggested a specific role for ABCB5^+^ MSCs in T-cell regulation in mammalian skin [10].

Indeed, in this study, following intravenous grafting to mice, syngeneic or fully MHC-mismatched ABCB5^+^ MSCs significantly inhibited murine alloantigen-dependent T-cell proliferation in mixed lymphocyte reactions and T-cell proliferation in response to mitogens, revealing that ABCB5^+^ MSCs possess the capacity to modulate primary T-cell-mediated immune responses in vivo [10]. In fully MHC-mismatched cardiac allotransplantation models, allogeneic ABCB5^+^ MSCs significantly prolonged allograft survival [10]. Characterization of the in vivo trafficking pattern of DiO-labeled ABCB5^+^ MSC grafts 10 days post-cardiac allotransplantation (day 17 post cell administration) revealed high frequencies in the skin and the thymus but low to absent levels in the other tissues examined, in line with the observed expression of molecules important for homing to the skin (CCR4, CCR10, E-selectin ligands) and thymus (CCR7, E-selectin ligands) [76,77,78] by ABCB5^+^ MSCs [10]. These findings suggested that ABCB5^+^ MSCs prolonged graft survival systemically, potentially by tolerizing thymic immune cells to alloantigens, rather than locally by interacting with allograft-infiltrating lymphocytes [10]. Indeed, intravenous administration of allogenic ABCB5^+^ MSCs resulted in increased percentages of splenic CD4^+^CD25^+^Foxp3^+^ regulatory T-cells (Tregs), consistent with 3-fold increased frequencies of intracardiac Foxp3^+^ Treg infiltrates in the cardiac allografts as compared with untreated control allograft recipient mice.

Remarkably, stable PD-1 knockdown induced by PD-1 short hairpin RNA reversed the inhibitory effects of ABCB5^+^ MSCs on T-cell proliferation, inhibited ABCB5^+^ MSC-dependent induction of Tregs, and attenuated ABCB5^+^ MSC-induced prolongation of cardiac allograft survival, confirming that these immunoregulatory effects of ABCB5^+^ MSCs are mediated, at least in part, through PD-1 [10]. Furthermore, administration of allogeneic PD-1-expressing ABCB5^+^ MSCs to PD-L1^−/−^ mice deficient in the PD-1 ligand PD-L1 [79] did not significantly alter Treg numbers as compared to untreated controls [10]. This observation indicates that efficient ABCB5^+^ MSC-dependent Treg induction requires interactions between PD-1 expressed on ABCB5^+^ MSCs and PD-L1 expressed on host cells [10]. Together, these studies showed that allogeneic ABCB5^+^ PD-1^+^ MSCs can exert, in a stem cell graft PD-1/recipient PD-L1-dependent fashion, significant in vivo inhibitory effects on alloreactive T-cell activation, along with induction of tolerogenic CD4^+^CD25^+^Foxp3^+^ Treg responses [10], providing a rationale for their potential therapeutic development in settings of unwanted allo-activation such as, for example, allograft rejection or graft-versus-host disease (GvHD).

#### 5.2.2. Effects on Neutrophils

Neutrophil granulocytes constitute a first line of defense against invading pathogens [80,81]. Upon activation by pathogen- or damage-associated molecular patterns [81,82], neutrophils produce high concentrations of antimicrobial reactive oxygen species (ROS) to create a toxic environment for microorganisms [82]. Moreover, ROS can further augment the antimicrobial response of neutrophils by triggering the release of granules, stimulating the production of pro-inflammatory cytokines, and inducing the formation of neutrophil extracellular traps (NETs) [82,83,84]. However, while a functioning neutrophil response plays an important role in protecting the organism from potentially life-threatening infections [81], overactivation of neutrophils, either by persisting stimuli or by impeded resolution, can contribute to the pathology of various inflammatory conditions [84] including, among several others, immune complex-mediated vasculitis [85,86] and chronic, non-healing wounds [86].

In skin sections from human vasculitis patients and from a murine immune complex-mediated vasculitis model of unbalanced neutrophil activation mimicking human vasculitis with unrestrained NET formation and severe tissue damage [87], immunostaining for ABCB5 and superoxide dismutase 3 (SOD3) revealed a significantly higher frequency of double-positive MSCs as compared with healthy controls [88]. In vitro, ABCB5^+^ MSCs suppressed ROS release and NET formation by human neutrophils activated with phorbol myristate acetate (PMA) [88]. Together, these observations suggest that endogenous ABCB5^+^ MSCs, in their physiological niche, mount an adaptive antioxidant response by SOD3 overexpression, to protect themselves from the threat of neutrophil oxidative burst, even though, at their low physiological numbers, they are not able to protect the skin from vasculitis and vasculitis-associated tissue breakdown [88]. In the same murine vasculitis model, intra-dermal injection of AT-MSCs significantly reduced the numbers of neutrophil elastase- and myeloperoxidase-positive neutrophils, NET formation, and vascular leakage via upregulation of SOD3 [88]. It seems reasonable to hypothesize that ABCB5^+^ MSCs, upon application in therapeutical doses, would also be capable of counteracting ROS release from overactivated neutrophils and preserving tissue integrity via upregulation of SOD3. However, at present this remains to be confirmed.

#### 5.2.3. Effects on Macrophages

Macrophages play an important role in skin tissue homeostasis as well as in host defense against invading pathogens and in response to injury. Displaying a high degree of plasticity, they can differentiate into pro-inflammatory (M1) or pro-regenerative (M2) macrophages to promote or suppress inflammation and modulate wound healing [89,90]. In normal tissue, macrophage recruitment, polarization and function are tightly regulated. During the physiological wound healing process, the transcriptome of wound macrophages undergoes complex changes, resulting in conversion from M1 to M2 phenotype [90]. In contrast, non-healing wounds are characterized by persistent high numbers of overactivated M1 macrophages releasing enhanced amounts of pro-inflammatory cytokines [90,91,92,93], which, in concert with proteases and ROS, induce a senescence program in resident fibroblasts and promote tissue degradation [94,95].

As observed with other MSC types [96,97], ABCB5^+^ MSCs can interact with macrophages in several ways, including influencing their recruitment to inflammatory sites, inducing their transition from M1 to M2 phenotype, and modulating their function.

Upon early, short-term (6 h) coculture with interferon γ (IFN-γ)/lipopolysaccharide (LPS)-activated M1 macrophages, ABCB5^+^ MSCs switch their own transcriptomic profile to genes and gene products involved in the mobilization of immune cells [98]. Among them are CXCL2, CXCL10, interleukin (IL)-1β and IL-6 [98], which have been shown to promote monocyte recruitment and differentiation into macrophages [97,99,100,101]. This suggests that in the early inflammatory phase, when the attraction of macrophages is essential to counteract microbial invasion and clean injured sites from debris, ABCB5^+^ MSCs can enhance inflammation and macrophage recruitment. In contrast, in conditions associated with sustained excessive macrophage infiltration into the skin, such as in the *Col7a1*^−/−^ NSG neonatal mouse model of recessive epidermolysis bullosa (RDEB), intravenous application of ABCB5^+^ MSCs can strongly decrease CD68^+^ macrophage skin infiltration, leading to significant amelioration of disease activity [102].

In an iron-overload mouse wound model mimicking the chronic inflammatory state of nonhealing human venous ulcers, which is characterized by persistent, unrestrained M1 macrophage activation [94], ABCB5^+^ MSCs injected intradermally around the wound edges of full-thickness excisional wounds localized within the wound bed in close association to endogenous murine macrophages [11]. This was followed by an increase in the numbers of CD206^+^ F4/80^+^ M2 macrophages in the wound tissue, while tumor necrosis factor (TNF)-α^+^ F4/80^+^ M1 macrophages became virtually absent, in sharp contrast to high numbers of TNF-α^+^ F4/80^+^ macrophages persisting in the wound margins of vehicle- or ABCB5^−^ fibroblast-injected iron-overload wounds [11]. Immune phenotyping of F4/80^+^ wound single-cell preparations confirmed an immunophenotype shift with downregulation of the M1 activation markers TNF-α, IL-12/IL-23p40 and inducible nitric oxide synthase (NOS2), while the M2 activation markers CD206, dectin-1 and arginase-1 were upregulated. In parallel, levels of the pro-inflammatory cytokine IL-1β and its downstream effector TNF-α in the wound bed tissue decreased, while anti-inflammatory IL-10 levels increased as compared with vehicle- or ABCB5^−^ fibroblast-injected wounds. These changes were associated with faster re-epithelialization, increased neovascularization, and improved tissue remodeling, which ultimately resulted in significantly accelerated wound closure [11]. Silencing of IL-1 receptor antagonist (IL-1RA) in ABCB5^+^ MSCs abrogated suppression of TNF-α^+^ F4/80^+^ macrophages, reversed IL-1β and TNF-α suppression and IL-10 upregulation, and abrogated accelerated wound closure, uncovering a causal role for IL-1RA secreted by ABCB5^+^ MSCS to abrogate M1 macrophage persistence and induce a phenotype shift to healing-promoting M2 macrophages in chronic wounds [11].

**Figure 4:**
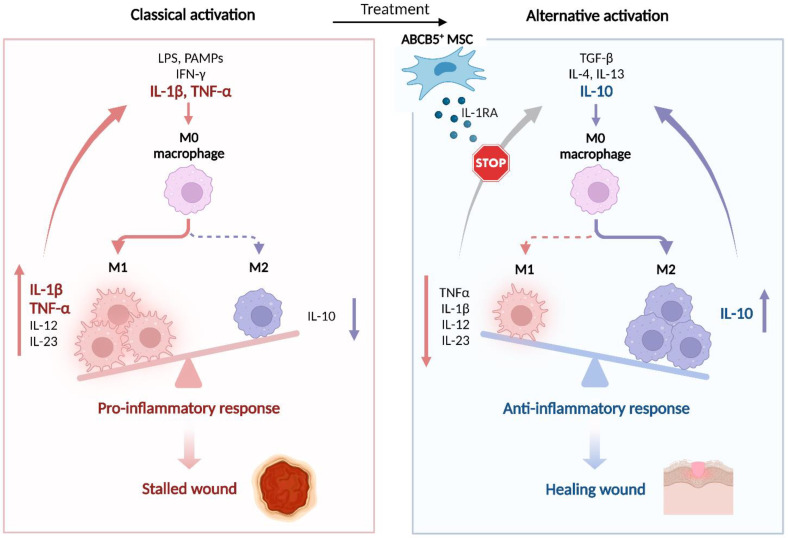
ABCB5^+^ MSCs trigger M1-to-M2 macrophage phenotype shift in chronic wounds. Chronic wounds are stalled in the inflammatory phase through IL-1β- and TNF-α-mediated persistent autocrine recruitment and activation of pro-inflammatory M1 macrophages (left panel). Upon therapeutic application (right panel), ABCB5^+^ MSCs block IL-1β signaling by adaptive release of IL-1RA, thereby breaking the vicious cycle of autocrine M1 macrophage activation. This shifts the balance from M1 macrophage-dominated inflammation to M2 macrophage-dominated healing-promoting tissue environment characterized by decreased levels of pro-inflammatory cytokines such as TNF-α, IL-12 and IL-23, and enhanced production of the anti-inflammatory cytokine IL-10. IFN-γ—interferon γ; IL—interleukin; LPS—lipopolysaccharides; PAMPs—pathogen-associated molecular patterns; TGF-β—transforming growth factor β. Adapted from “The Key Role of Neuroinflammation in Neurodegenerative Diseases”, by BioRender.com (2022). Retrieved from https://app.biorender.com/biorender-templates.

While unstimulated ABCB5^+^ MSCs in culture do not readily produce IL-1RA, they release high amounts of IL-1RA when stimulated with IFN-γ and LPS. The IL-1RA concentrations were observed to be even higher upon coculture of ABCB5^+^ MSCs with IFN-γ/LPS-activated M1 macrophages for 24 h [11]. Following injection of ABCB5^+^ MSCs into the wound edges in the iron-overload model, IL-1RA expression was shown to occur in ABCB5^+^ MSCs and detected in the wound lysate, implying an adaptive release of IL-1RA by ABCB5^+^ MSCs in response to the inflammatory environment [11]. Loss of the capacity of IL-1RA-silenced ABCB5^+^ MSCs to induce a wound macrophage M1-to-M2 phenotype shift was found to be associated with a reversal of TNF-α and IL-1β suppression and IL-10 upregulation in wound lysates, indicating that IL-1RA adaptively released from ABCB5^+^ MSCs upon stimulation at the wound sites suppresses IL-1 signaling and the downstream effector TNF-α, while inducing anti-inflammatory IL-10 [11]. As TNF-α and IL-1β have been shown to mediate M1 macrophage recruitment and activation in an autocrine manner [94,103], the capacity of ABCB5^+^ MSCs to shift the M1 macrophage prevalence towards an M2 phenotype has therefore been attributed to IL-1RA-mediated interruption of a vicious cycle of unrestrained autocrine M1 macrophage activation [11] (Figure 4).

Comparative coculture experiments have revealed that IL-1RA secretion by ABCB5^+^ MSCs in response to IFN-γ/LPS-activated macrophages is reduced when the macrophages are separated from the MSCs by a transwell chamber, which prevents cell-cell contact between both cell types, as compared with direct coculture [104]. This suggests that ABCB5^+^ MSCs sense macrophages in their environment via both paracrine and direct, cell contact-dependent signaling [104]. Interestingly, in direct coculture with macrophages, but not in the transwell system, ABCB5^+^ MSCs adaptively upregulate expression of secreted phosphoprotein 1 (SPP1), the gene encoding osteopontin. Moreover, silencing of SPP1 in ABCB5^+^ MSCs impeded IL-1RA upregulation and induction of the M1-to-M2 phenotype shift [98]. This suggests that the SPP1 product osteopontin upregulated by ABCB5^+^ MSCs in response to direct cell contact with macrophages plays a key role in the regulation of the IL-1RA-mediated immunomodulatory effects of ABCB5^+^ MSCs on macrophages [98].

Apart from their actions on M1 macrophages, ABCB5^+^ MSCs can also modulate distinct functions of M2-polarized macrophages such as their phagocytic activity, at least in vitro [98]. While, in normal wound healing, phagocytosis of apoptotic neutrophils by macrophages is a key step to resolution of inflammation and initiation of tissue formation [105], impaired phagocytic activity of macrophages has been described to play a critical role in the pathogenesis of impaired wound healing such as in diabetes or during aging [106,107]. It may thus be noted that co-culture with ABCB5^+^ MSCs enhanced the phagocytic activity of M2-polarized macrophages in an FITC-dextran phagocytosis assay [98].

#### 5.2.4. Anti-Infection

In contrast to the strong immunosuppressive reactions of ABCB5^+^ MSCs on allogeneic responses and persistent sterile inflammatory processes, ABCB5^+^ MSCs appear not to inhibit immune cell activities to the same extent in the presence of infectious agents, but instead may facilitate the antimicrobial defense [91]. Whereas, in an uninfected microenvironment, ABCB5^+^ MSCs suppress ROS release and NET formation by activated human neutrophils [88] (see Section 5.2.2. Effects on Neutrophils), upon challenging with LPS, a wall component of gram-negative bacteria, ABCB5^+^ MSCs undergo a toll-like receptor (TLR)-4/nuclear factor (NF)-κB-dependent fundamental transcriptomic shift to a microbicidal transcriptome characterized by significant upregulation of neutrophil-activating chemokines, which results in increased NET formation after co-culture with activated neutrophils [108]. This suggests that ABCB5^+^ MSCs can sense bacterial infection in their environment, which promotes them to adopt an unusual pro-inflammatory phenotype that, for the sake of tissue protection from invading pathogens, stimulates activation of neutrophils [109].

Notably, the capacity to adaptively respond to the pathogen signal LPS differs between ABCB5^+^ MSCs derived from young (<30 years) versus old (>60 years) individuals: While ABCB5^+^ MSCs from young and old donors displayed a similar quantitative expression and time-dependent regulation of the LPS sensing receptor TLR-4, old donors exhibited a significantly delayed back-regulation of NF-κB translocation to the nucleus of LPS-primed ABCB5^+^ MSCs and a markedly reduced NET formation as evidenced by decreased neutrophil elastase activity in cocultures of LPS-primed ABCB5^+^ MSCs and PMA-activated neutrophils as compared with young donors [108]. These observations suggest that ABCB5^+^ MSCs derived from older donors cannot raise adaptive responses to infections cues in their microenvironment to the same extent as ABCB5^+^ MSCs from younger individuals [108].

### 5.3. Trophic Effects

In response to signals associated with tissue injury, MSCs release bioactive trophic factors that stimulate neighboring cells to start repairing damaged tissues through angiogenesis, remodeling of the ECM and/or supporting parenchymal cells [3,57,110].

#### 5.3.1. Angiogenesis

Among the most potent environmental factors that stimulate MSCs to secrete proangiogenic molecules is hypoxia [111], which, in injured or diseased tissues, can result from an impaired blood supply due to blood vessel damage. When exposed to hypoxic culture conditions, ABCB5^+^ MSCs activate the HIF-1α pathway, which is followed by upregulation of transcription of several downstream genes, including factors that reduce cellular oxygen consumption, the proangiogenic factor VEGF, and the proangiogenic receptors VEGFR-1 (Fms-like tyrosine kinase-1) and Tie-2 [68]. Quantitative VEGF mRNA analysis revealed 4-fold upregulation of VEGF transcription, which was followed by a strong increase in VEGF protein secretion [68]. The factor VEGF is a vital paracrine mediator in the signaling cascade to increase angiogenesis via stimulating proliferation, migration, differentiation, and survival of adjacent endothelial cells, and is thereby integral to tissue restoration in injury, ischemia, and wound healing [112,113].

In a mouse model of surgically induced hind limb ischemia, intramuscular injection of ABCB5^+^ MSCs significantly increased the number of CD31^+^ (endothelial-lineage) cells in the ischemic muscles, enhanced the proliferation of capillaries, and markedly accelerated perfusion recovery as measured by real-time laser doppler blood perfusion imaging [68]. This demonstrated that the proangiogenic actions of ABCB5^+^ MSCs can translate into an increase in vascularization and improvement of defective blood circulation in vivo.

Secretome analyses of unstimulated ABCB5^+^ MSCs have uncovered a significant enrichment of the proangiogenic ribonuclease angiogenin [114]. Angiogenin is a key regulator of blood vessel homeostasis, through both maintenance of endothelial cell self-renewal and stimulation of new vessel growth [115]. Conversely, biopsies from human non-healing wounds such as chronic venous leg ulcers and chronic diabetic foot ulcers have depicted a significantly decreased angiogenin expression as compared with biopsies obtained from acute wounds [114]. Intradermal injection of ABCB5^+^ MSCs at the wound edges in a *db/db* mouse diabetic wound model significantly enhanced angiogenesis in the wound tissue and accelerated wound healing as compared with vehicle- and fibroblast-injected *db/db* wounds [114]. Silencing of angiogenin in ABCB5^+^ MSCs abrogated these effects, identifying a key role for angiogenin secreted by ABCB5^+^ MSCs at the wound site to stimulate new vessel formation in diabetic wounds [114]. This was supported in an in vitro tube formation assay where the addition of ABCB5^+^ MSCs, in contrast to angiogenin-silenced ABCB5^+^ MSCs, to cocultures of HUVECs and *db/db* murine dermal fibroblasts rescued the impaired diabetic fibroblast-dependent tube formation [114].

#### 5.3.2. ECM Remodeling

Being a highly dynamic structure, the ECM continuously undergoes regulated remodeling involving deposition, modification, and enzymatic degradation of ECM components. A tight orchestration of these processes is essential for normal tissue function and organ homeostasis [116,117,118]. In the skin, ECM components are integral in each stage of wound healing [119,120,121], and ECM dysfunctions are associated with impaired cutaneous wound healing and abnormal scarring [122]. One of the most crucial ECM components is collagen, which plays central roles in the regulation of several key steps in wound healing such as platelet aggregation, inflammation modulation, angiogenesis, granulation tissue and scar tissue formation, and re-epithelization [123].

The ABCB5^+^ MSCs injected into the wound edges of excisional wounds in an iron-overload mouse wound model mimicking nonhealing human venous ulcers [94] markedly improved tissue remodeling with increased maturation and improved organization of collagen fibers similar to acute wounds, which was associated with reduced scar formation and improved tensile strength of the scar tissue [11]. In *db/db* mice, which display severely reduced collagen deposition in the dermis as compared with wildtype mice, intradermal injection of ABCB5^+^ MSCs enhanced collagen deposition in full-thickness wounds [114]. This effect was abrogated by silencing of angiogenin, indicating a critical role of angiogenin in ABCB5^+^ MSC-mediated wound remodeling in the diabetic organism [114]. The observation that in the *db/db* wound model repetitive injections of ABCB5^+^ MSCs were less efficient in accelerating wound closure and induced a stronger fibrotic response as compared with wounds that were injected only once suggests that an appropriate timely orchestration is important to achieve an optimal response [114].

The above-mentioned studies have not addressed whether the observed effects of ABCB5^+^ MSCs on the ECM represented direct effects or were mediated indirectly through modulating macrophage or endothelial cell function. However, evidence supporting a potential ability of ABCB5^+^ MSCs to directly contribute to ECM remodeling has come from secretome analyses revealing that ABCB5^+^ MSCs, in contrast to BM-MSCs, express the basement membrane proteins collagen type VII and laminin-332 (α3 and β3 subunits) [104], both of which are critical for cutaneous wound healing [124]. Whether ABCB5^+^ MSCs are capable of efficiently depositing these proteins in the ECM remains, however, to be elucidated [104].

In contrast, in situations of excessive, detrimental ECM deposition, ABCB5^+^ MSCs have appeared to be capable of reducing the amount of deposited collagen. In the *Mdr2^−/−^* mouse model of established cholestatic liver fibrosis, intravenously injected ABCB5^+^ MSCs significantly reduced collagen deposition, particularly in the periportal region [125]. The observed reduction was not associated with changes in liver tissue expression of αSMA and smooth muscle protein 22α, marker proteins of activated hepatic stellate cells and portal fibroblasts, which are the major contributors to cholestatic liver fibrosis [126,127]. This suggested that the reduction in liver collagen deposition likely resulted from increased ECM degradation rather than decreased fibrogenesis [125].

#### 5.3.3. Effects on Parenchymal Cells

Although the precise endogenous roles of MSC populations residing in various body tissues is incompletely understood, consistent evidence suggests that they play crucial roles in organ homeostasis and the support of parenchymal cells [110,128]. Consequently, MSCs might open up therapeutic options in the regeneration of parenchymal organs following tissue injury [57].

The ABCB5^+^ MSCs have been studied in polycystic kidney disease (PKD), a group of inherited nephropathies characterized by the formation of multiple fluid-filled cysts in the kidney, which distorts the renal architecture and severely impairs filtration function [129,130]. In the PKD/Mhm (Cy/+) rat model of the most common PKD type, i.e., autosomal dominant PKD (ADPKD) [131], systemic infusions of ABCB5^+^ MSCs elicited in the kidney cells a reversion of metabolic reprogramming from ADPKD-typical upregulation of alternative pathways of energy production such as glycolysis to oxidative phosphorylation, citrate cycle, gluconeogenesis, and pyruvate metabolism pathways [132]. Besides metabolic reprograming, treatment with ABCB5^+^ MSCs resulted in downregulation of the PI3K-Akt signaling pathway [132], overactivation of which plays a significant role in PKD cyst formation [133,134], and modulation of several additional pathways involved in apoptosis, cellular senescence, focal adhesion and inflammation [132]. The observed gene profile changes were associated with decreased numbers of renal cysts and apoptotic (TUNEL^+^) and proliferative (Ki-67^+^) cells in kidney tissue sections and translated into improvements in renal function as evidenced by enhanced glomerular filtration rate and reduction in proteinuria and albuminuria [132]. Therefore, ABCB5^+^ MSCs might represent a potential candidate therapeutic for reducing cyst growth and improving kidney function in PKD.

## 6. Therapeutic Use

### 6.1. Feasibility of Allogeneic Use

Mesenchymal stem cell therapy requires safe and efficacious administration of the cell product. First attempts of therapeutic MSC administration used autologous MSCs, derived from the patients themselves, and autologous MSCs are still used in most clinical trials [135]. For the first clinical trial of ABCB5^+^ MSCs, the cells were derived from small skin biopsies of the patients and expanded ex vivo, before being topically applied onto chronic, standard therapy-refractory venous ulcers (CVUs) [39]. While the clinical outcome of a median 63% wound size reduction at 12 weeks from baseline, also associated with early pain reduction, substantiated the view that autologous ABCB5^+^ MSCs could deliver a clinically relevant wound closure strategy, the autologous approach emerged associated with several drawbacks owing to the labor- and time-consuming manufacturing process, which was carried out for each patient individually. This was associated with comparably high costs and weeks- or monthslong delays until the treatment could be started, and generated only limited numbers of cells, which neither enabled treatment of larger wounds [39] nor would allow for higher cell number-requiring potential systemic therapy approaches. In addition, candidate patients for MSC therapies are often of advanced age and frequently affected by comorbidities. Unfortunately, age and disease state can negatively affect the number and functionality of MSCs [136]. The ABCB5^+^ MSCs, as outlined above (see Section 4.2. Cutaneous Regeneration and Wound Healing), frequently underlie age-dependent declines in the number of cells present in situ [44,47] and can accumulate DNA damage and enhanced apoptosis [45] as well as changes in the differentiation potential [45,46]. Moreover, diabetes, at least in mice, is associated with reduced numbers of ABCB5^+^ MSCs along with profound changes in the dermal stromal niche [114].

These hurdles associated with the use of autologous MSCs prompted additional research on allogeneic treatment strategies. In the allogeneic setting, ABCB5^+^ MSCs can be obtained from young, healthy donors and manufactured as a ready-to-use product of consistent quality [12]. The generally low immunogenicity of resting MSCs, owing to only low or modest expression of MHC class I molecules and lack of MHC class II and co-stimulatory molecules required for full T-cell activation [137,138,139], and their immunosuppressing capacities (see Section 5.2. Immunomodulation) contribute to a lower immune rejection response elicited by transplanted allogeneic MSCs compared to other cell types [140,141]. Therefore, although previous studies that controlled for MHC haplotype of donors and recipients suggested that MHC-mismatched MSCs may not be immune-privileged [141,142,143] and that about 11.5% of patients may develop donor-specific antibodies upon administration of allogenous MSCs [144], the transplantation of allogeneic MSCs typically requires no immunosuppressive treatment of the recipient.

A body of studies that directly compared immune-modulatory and regenerative efficacies of allogeneic vs. autologous MSCs in immunocompetent animals or in humans have confirmed that allogeneic MSCs are not inferior to autologous MSCs [145,146,147,148,149,150,151]. This applies also to ABCB5^+^ MSCs: Comparison of clinical trial results using either autologous, patient-derived or allogeneic, donor-derived ABCB5^+^ MSCs to treat chronic, treatment-refractory CVUs reveals that allogeneic treatment achieved a median wound size reduction from baseline at 12 weeks of 76% (full analysis set) and 78% (per-protocol set) with 20% and 22%, respectively, of wounds fully closed [152], as compared with 63% wound size reduction and 17% full wound closure rate in the autologous setting [39]. Moreover, in fully MHC-mismatched cardiac allotransplantation models, allogeneic (donor-strain or third party-strain) ABCB5^+^ MSCs profoundly prolonged graft survival, whereas treatment with syngeneic (recipient-strain) ABCB5^+^ MSCs showed only modest prolongation of graft survival, indicating that prolonged enhancement of cardiac allograft survival by treatment with ABCB5^+^ MSCs even depends on MSC-dependent allogeneic tolerogenic stimulation [10].

### 6.2. Homing and Engraftment

**Figure 5:**
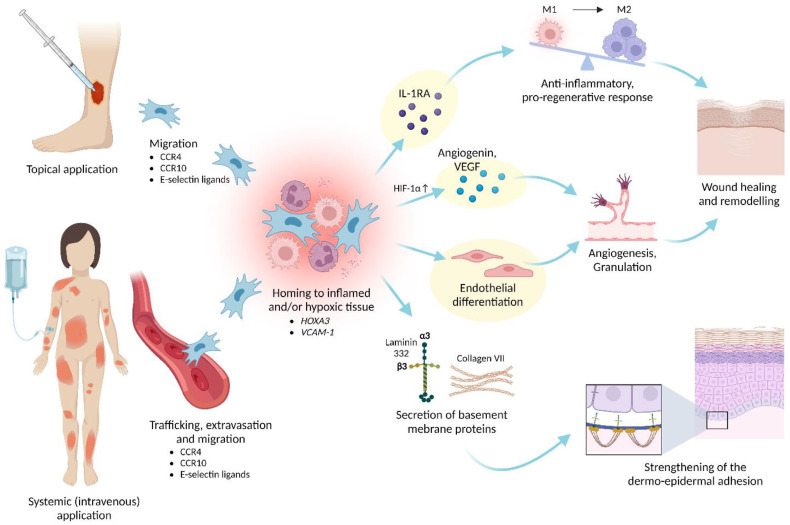
Clinical use and effects of ABCB5^+^ MSCs. Upon topical or systemic administration, ABCB5^+^ MSCs migrate and home to injured, inflamed and/or hypoxic tissues to exert anti-inflammatory, pro-angiogenic and trophic responses that facilitate wound healing and enhance skin integrity. The mode-of-action pathways that are utilized for routine potency testing of the MSC-based medicinal product are highlighted in yellow. HIF-1α—hypoxia-inducible factor 1α; *HOXA3*—homeobox A3; M1—M1 macrophage; M2—M2 macrophage; *VCAM-1*—vascular adhesion molecule 1. Created with BioRender.com.

When tissues are damaged, endogenous MSCs migrate to the site of injury to locally trigger mechanisms that promote regeneration [153]. The resultant rationale behind therapeutic application of MSCs is that transplanted MSCs would also migrate and home to the damaged tissues [154] (Figure 5).

Upon local administration in spatial proximity to a lesion, the MSCs can be directed to the target tissue via a chemokine gradient released from the site of injury [155]. In a chronic wound model characterized by excessive pro-inflammatory macrophage activation [94], human ABCB5^+^ MSCs that had been intradermally injected around the wound edges localized within 2 days in the wound bed to close proximity with endogenous macrophages [11]. This was shown to be a prerequisite for paracrine suppressive activity of ABCB5^+^ MSCs directed at overactivated wound macrophages [11].

Systemic administration of MSCs offers the advantage that grafted cells can migrate to multiple and/or difficult-to-access sites of tissue injury. Upon injection into the systemic circulation, MSCs must undergo a multi-step process that involves travelling in the vascular system and egressing from the circulation in a lesion’s vicinity followed by migration through the interstitium towards a target site [154,155]. In these regards, intravenously administered mouse dermal ABCB5^+^ MSCs have emerged capable of efficiently homing to mouse skin and thymus [10]. This tropism pattern is consistent with mouse ABCB5^+^ MSC expression [10] of molecules important for homing to the skin (CCR4, CCR10, E-selectin ligands) and thymus (CCR7, E-selectin ligands) [76,77,78].

Generally, only small percentages of systemically administered MSCs reach the target tissues, which is considered a major bottleneck in reaching the full therapeutic potential of systemic MSC therapies [154]. It is therefore noteworthy that the capacity of systemically grafted human ABCB5^+^ MSCs to home to NSG mouse skin wounds significantly exceeded that of directly compared BM-MSCs [104]. The superior skin homing properties of ABCB5^+^ MSCs over BM-MSCs might relate to their physiological derivation from the skin, as well as to the higher expression of the homeobox gene *HOXA3*, a master coordinator of wound repair through regulating migration of endothelial and epithelial cells [156,157], and of vascular adhesion molecule 1 (*VCAM-1*) by ABCB5^+^ MSCs compared to BM-MSCs [104].

It is still controversial whether therapeutically applied MSCs, beyond migrating to their target sites to exert transient effects, also possess longer-term engraftment potential. Nevertheless, some observations point to a distinct ability of ABCB5^+^ MSCs to engraft in host target tissues. Upon topical application of human ABCB5^+^ MSCs to full-thickness excisional skin wounds in NSG mice, dose-dependent detection of human CD31 in the wound bed on day 13 indicated that the transplanted cells had persisted and differentiated into CD31^+^ endothelial-lineage cells in the recipients’ wounds for at least 13 days [39]. Furthermore, immunohistochemical detection of ABCB5-expressing cells amongst CVU wound-covering cell debris on day 23 following topical application of autologous ABCB5^+^ MSCs suggested persistent engraftment also in human wounds, though thus far only in the autologous setting [39]. Upon systemic, intravenous injection, engrafted mouse ABCB5^+^ MSCs persisted in mouse skin and thymus across fully allogeneic barriers for at least 17 days [10]. Moreover, intravenously grafted human ABCB5^+^ MSCs persisted in NSG mouse full-thickness excisional wounds for at least 14 days, exhibiting superior engraftment capacity compared to intravenously grafted human BM-MSCs [104]. The latter observation opens up potential options for MSC-based central tolerization strategies of thymic immune cells to alloantigens, e.g., in solid organ transplantation [10], the induction of splenic regulatory T-cells [10], e.g., in GvHD, and for the systemic treatment of generalized skin diseases, including severe genodermatoses such as epidermolysis bullosa [104] (see Section 7. Clinical Iindications).

### 6.3. Product Quality

#### 6.3.1. Homogeneity

The ATMPs based on living cells are inherently prone to heterogeneity because the cells’ gene and expression profiles may vary considerably depending on variations in donor characteristics as well as methods and conditions of cell expansion, cell isolation and product formulation [8,158,159,160]. In the ABCB5^+^ MSC manufacturing process, careful donor selection utilizing rigorous in-/exclusion criteria regarding age and health state, strict definitions of all production steps and in-process controls at every stage of production [12], and, not at least, the use of a unique cell surface marker (ABCB5) specifically selecting for a reproducibly potent MSC subpopulation [10,11] are critical tools to minimize potential fluctuations.

Indeed, during process validation, comparative gene expression analyses revealed only rare variations between donors as well as between different passages within donors, which indicates high intra- and inter-donor homogeneity of the expanded cells [39]. As evaluated in periodic GMP product quality reviews [12], homogeneity of GMP-compliantly manufactured ABCB5^+^ MSCs is reliably achieved by close monitoring and continuous refinement of each process step and mandatory release tests ensuring identity, purity, and biological activity of the produced cells. Specified, validated acceptance criteria guarantee that each released cell batch contains ≥90% ABCB5^+^ cells (actually 97.7 ± 1.9%) and ≥90% CD90^+^ cells (actually 99.5 ± 0.5%) at ≥90% cell vitality (actually 98.6 ± 0.6%) and ≥90% cell viability (actually 99.5 ± 0.6%) (means ± SD of 66 cell batches derived from seven donors [12].

#### 6.3.2. Potency

A vital component of the quality assessment of cell therapy products is the evaluation of the cells’ actual biological functionality using appropriate tests that predict their clinical effectiveness [161,162]. A validated potency assay with predefined acceptance criteria guarantees that the product exerts a certain intended effect at a specific dose and ensures that the treated patient receives a potent therapy product [163]. Considering that ABCB5^+^ MSCs exert their therapeutic effects through multiple pathways induced upon interaction with the host microenvironment (detailed in Section 4. Biopharmacological Modes of Action), three potency assays have been integrated into the manufacturing process to reflect the most clinically relevant biological modes of action of ABCB5^+^ MSCs (Figure 5):

1. Secretion of IL-1RA after coculture with M1-polarized macrophages [12,39] as a predictive measure of the anti-inflammatory potency in M1 macrophage-dominated inflammatory milieus (see Section 5.2.3. Effects on Macrophages).
2. Secretion of VEGF under hypoxic culture conditions [12,68] as a predictive measure of the pro-angiogenic bioactivity in ischemic tissue environments (see Section 5.3.1. Angiogenesis).
3. Tube formation on gel matrix [12,68] as a predictive measure of the endothelial differentiation capacity (see Section 5.1. (Trans-)Differentiation).

These three potency assays are an integral component of the release test panel for human skin-derived ABCB5^+^ MSCs, and only batches that have met the specified, validated acceptance criteria for each assay are released [12].

### 6.4. Safety

#### 6.4.1. Product Safety

Ex vivo expansion entails enhanced cell division rates in an artificial environment lacking the physiological mechanisms of negative selection and clearance of altered cells that are active in the whole organism [164]. Therefore, in order to facilitate early detection of signals of non-physiological growth behavior during cell expansion that might indicate potential deleterious effects on cell biology including tumorigenic transformation, several in-process controls monitoring cell morphology, contact inhibition, time between passages and cell cycle phase distribution are vital components of the routine ABCB5^+^ MSC manufacturing process [12].

To ensure sterility and purity of the manufactured cell products, specified validated control procedures guard, at each production step, against potential contamination of the cultures, cellular intermediate and final products with infectious agents (including aerobic and anaerobic bacteria and fungi, mycoplasma, and endotoxins) and residual impurities [12]. Only cultures and batches that meet specified release criteria ensuring product sterility and purity are released for further processing, cryostorage, or delivery [12].

#### 6.4.2. Preclinical Safety Profile

The preclinical safety profile of GMP-compliantly produced, reconstituted (ready-to-use) ABCB5^+^ MSCs has been determined in a Good Laboratory Practice-conforming preclinical in vivo study program following the recommendations of the European Medicines Agency [161] addressing all relevant aspects of biosafety [165]. All studies were performed in severely immunocompromised (NOD scid or NSG) mice to prevent rejection of the administered cells in the xenogeneic host. In these mice, subcutaneously injected ABCB5^+^ MSCs did not significantly migrate to other tissues and organs, indicating confinement of the cells to the application site following local administration. Systemic (intravenous) infusion at a concentration intended for use in clinical trials (1 × 10^7^ cells/mL [12,166]) was well tolerated without any clinical signs or mortality indicative of clinically relevant pulmonary embolus formation by cells mechanically entrapped in the lungs. Repeated subcutaneous or intravenous injections neither provoked any signs of human cell-related tumor development, ectopic tissue formation or micrometastases, nor did it elicit any signal indicative of ABCB5^+^ MSC-related toxicity regarding mortality, clinical signs, body weight development, food consumption, ophthalmological examination, urine analysis, hematology, blood chemistry, blood coagulation, and macro-pathological and histopathological examination. Together, these data demonstrated a favorable biosafety profile of GMP-manufactured ABCB5^+^ MSCs in terms of distribution to non-target tissues, toxicity, ectopic tissue formation, or tumor development [165].

In intramuscular local-tolerance studies, injection of ABCB5^+^ MSCs into the thigh muscles of NOG mice resulted in a slight increase in microscopically detectable inflammatory and detectable processes in the muscular tissue when compared to vehicle-treated animals at 1 week, whereas at 4 weeks no differences between cell- and vehicle-injected thigh muscles were detectable [165].

Several lines of evidence of liver safety of ABCB5^+^ MSCs have come from studies investigating the effects of ABCB5^+^ MSCs, delivered to the liver via the intrasplenic route to bypass pulmonary entrapment, in an *Pfp/Rag2*^−/−^ immunodeficient mouse model of liver regeneration after partial (one third) liver resection [125,167]. In this model, GMP-manufactured ABCB5^+^ MSCs did not augment the increase in serum transaminases that occurred following partial hepatectomy over controls at week 7 after MSC application [167]. In addition, ABCB5^+^ MSC did not impact on physiological lipid accumulation, hepatocyte proliferation rate, physiological zonal distribution of metabolism markers (i.e., periportal expression of E-cadherin, and perivenous expression of glutamine synthetase and cytochrome P450 2E1) during seven weeks of liver regeneration [167]. Moreover, ABCB5^+^ MSCs did not induce toxicity regarding liver fibrosis (as assessed by collagen deposition and expression of *Timp1*, the gene encoding tissue inhibitor of metalloproteinases 1), inflammation (as assessed by expression of the pro-inflammatory cytokines Il1B and Il6 in liver tissue) or hepatocellular destruction (as assessed by expression of caspases 3 and 9 in liver tissue) as compared with vehicle-treated animals at week 7 after MSC application [103].

#### 6.4.3. Safety Data from Clinical Trials

At present, the clinical safety of allogenic ABCB5^+^ MSCs has been investigated in three completed phase I/IIa clinical trials (see Section 7. Clinical Indications) [68,152,166]. Overall, 54 adult patients presenting with chronic wounds received, in total, 92 topical doses of 1–2 × 10^6^ ABCB5^+^ MSCs per cm^2^ wound surface area [68,152], and 16 patients aged 4–36 years suffering from RDEB received 46 intravenous infusions of 2 × 10^6^ ABCB5^+^ MSCs per kg body weight in total [166].

Treatment-related adverse events were reported with 3 of 92 (3.3%) topical and 3 of 46 (6.5%) of intravenous applications (Table 3). Treatment-related AEs reported from topical treatment were related to the treated wounds and were of mild or moderate severity. None of these events were serious, and all resolved without sequelae [152]. Treatment-related AEs reported from intravenous treatment were one mild lymphadenopathy and two severe hypersensitivity events, which may have resulted from immunological sensitization following the previous ABCB5^+^ MSC infusion. These latter two events were considered serious; however, the affected patients recovered without sequelae shortly after withdrawal of treatment [166]. Given that the risk of hypersensitivity reactions can likely be reduced in future treatments by monitoring potential induction of anti HLA antibodies and premedication with antihistamines, the Trial Data Monitoring Committee evaluated the potential risk of hypersensitivity reactions as being justified by the anticipated treatment benefits for RDEB patients [166]. Together, the clinical experience to date indicates a favorable safety profile of the cell product with few mild and/or manageable adverse events.

## 7. Clinical Indications

The origin of ABCB5^+^ MSCs from the skin [10,11], the essential role of endogenous ABCB5^+^ MSCs in normal cutaneous wound healing [47], and the outstanding capability of locally and systemically administered ABCB5^+^ MSCs to home to the skin and to skin wounds [10,11,104] gave reason to study the immunomodulatory and regenerative properties and to develop the therapeutic potential of ABCB5^+^ MSCs in the topical and systemic treatment of non-healing cutaneous wounds (Figure 5).

Normal wound healing is a complex dynamic process of well-orchestrated interactions between cytokines, chemokines, growth factors and various resident and recruited cells, which proceeds through successive, partially overlapping stages, namely hemostasis, inflammatory, proliferative and remodeling phases [92,168,169,170]. Disturbances in the temporal and spatial regulation of these interactions result in stalling of the healing process and the development of painful and distressing chronic wounds [92,169,170]. Because the prevalence of chronic wounds continues to rise with the growing frequency of significant predisposing factors such as vascular diseases, diabetes and obesity in an increasingly aging population [171], there is an urgent need for advanced therapies to treat wounds that do not sufficiently respond to current standards of care.

### 7.1. Chronic Venous Ulcers

Although venous leg ulcers can often be successfully treated, a considerable proportion becomes chronic [172,173], with reported healing failure rates ranging from roughly 40% to 85% at 12 weeks and still from 13% to 40% at 1 year of treatment [152]. From a pathophysiological perspective, CVUs remain stalled in the inflammatory state of the wound healing cascade, owing to defective transition of pro-inflammatory M1 to granulation-promoting M2 macrophages [90]. Persistent high numbers of overactivated M1 macrophages release excessive amounts of pro-inflammatory cytokines including IL-1β and TNF-α, which, in concert with proteases and ROS, induce a senescence program in wound fibroblasts and ultimately lead to tissue degradation [94,95]. Moreover, IL-1β and TNF-α drive a vicious cycle of autocrine M1 macrophage recruitment and activation, which arrests the wound in a non-healing state [90,91,94] (Figure 4).

In an NSG mouse full-thickness excisional wound model for CVUs, in which iron-overloaded macrophages release elevated amounts of TNF-α [94], human ABCB5^+^ MSCs injected into the dermis around the wound edges migrated into the wound bed in close proximity to wound macrophages and induced a macrophage M1-to-M2 immunophenotype shift [11] (Figure 4). This conversion was associated with faster re-epithelialization, increased neovascularization, and improved tissue remodeling, which significantly accelerated wound closure and improved scar tissue quality as compared with wounds treated with vehicle or donor-matched ABCB5^−^ dermal fibroblasts [11]. Silencing of IL-1RA in ABCB5^+^ MSCs abrogated these effects, uncovering a causal role for IL-1RA in ABCB5^+^ MSC-mediated promotion of wound healing [11].

The wound healing-facilitating effects observed in the CVU mouse model [11] and in an autologous first-in-human clinical trial of ABCB5^+^ MSCs in CVU patients [39] (see Section 6.1. Feasibility of Allogeneic Use) paved the road to a multicentric, single-arm phase I/IIa trial of allogeneic ABCB5^+^ MSCs in CVU patients whose wounds had emerged as resistant to standard care. This was ensured by including only patients whose wounds had failed to improve within 3 months or to heal within 12 months of optimal phlebological treatment before enrolment and had not changed by ≥25% in wound size during a subsequent 4-week standard-of-care period before the cell treatment was started [152]. About two thirds (21 out of 31) of these highly treatment-refractory patients responded to one or two topical administrations of ABCB5^+^ MSCs, achieving a median wound size reduction of 87%, with 29% (6 of 21) of wounds having fully closed [152].

Because the median time to full wound closure was not reached within the 12-week efficacy follow-up, it might be expected that the full closure rate would increase further if the follow-up period was extended. Another question to be answered is whether the wound healing outcome could be increased further by using higher cell doses, given that a preclinical dose-selection study in a mouse acute wound model revealed a dose-dependent effect of ABCB5^+^ MSCs on wound size reduction [39]. At present, these hypotheses are being studied in a larger, randomized controlled multicentric phase IIb trial with a dose-ranging design and an extended (18 weeks) efficacy follow-up period (NCT04971161). At the time of manuscript revision, 68 patients have been treated, of which 40 patients have passed the efficacy follow up and 12 patients have completed the 10-month safety follow up.

### 7.2. Diabetic Foot Ulcers

Diabetic foot ulcers (DFUs) represent one of the most common and potentially serious complications of diabetes mellitus. While rapid healing is highly desired to improve quality of life and to prevent potential life-threatening complications such as infections and amputations, DFUs often respond only poorly to standard-of-care therapy, with reported healing failure rates of roughly 40–80% at 12 weeks and still 15–70% at 1 year of treatment [68]. Dysfunctional wound healing in diabetes is closely linked to a sustained inflammatory disposition associated with a persistent IL-1β-driven prevalence of pro-inflammatory M1 macrophages [103,174,175] in concert with impaired cellular responses to tissue hypoxia due to deficient HIF-1α-dependent upregulation of VEGF and other angiogenic growth factors by local fibroblasts and endothelial cells [176,177,178,179] resulting in a decreased amount of nascent microvasculature [180,181].

Physiologically, ABCB5^+^ MSCs were suggested to play an essential role in normal skin wound healing through regulation of a proangiogenic pAkt/HIF-1α/VEGF cascade [47] (see Section 4.2. Cutaneous Regeneration and Wound Healing). However, the number of ABCB5^+^ MSCs was found severely reduced in the skin of diabetic *db/db* mice as compared with wildtype mice [114]. Considering the potential of ABCB5^+^ MSCs to induce in macrophages the transition from the M1 to the M2 phenotype (see Section 5.2.3. Effects on Macrophages) and to promote angiogenesis (see Section 5.3.1. Angiogenesis), it is likely that a reduction in or the dysfunction of dermal ABCB5^+^ MSCs contribute to reduced angiogenesis and wound healing observed in diabetes. Conversely, intradermal injection of ABCB5^+^ MSCs at the edges of full-thickness incisional wounds in *db/db* mice substantially enhanced wound closure and partly restored reduced collagen deposition [114].

Building upon these observations, ABCB5^+^ MSCs have been investigated as a potential option for adjunctive treatment of human DFUs. In a multicentric, single-arm phase I/IIa clinical trial, patients suffering from standard treatment-refractory neuropathic plantar DFUs received one or two topical applications of allogeneic ABCB5^+^ MSCs [68]. The treatment elicited an early acceleration of wound healing, becoming statistically significant already at 2 weeks (median surface area reduction from baseline of 31%). At the end of the 12-week follow-up period, 17 of 23 (74%) of the patients had responded to treatment, achieving a mean reduction in wound surface area of 67%, with 35% (6 of 17) of wounds having fully closed. Remarkably, in the preceding ≥6-week screening period with standard care alone, in about half of the patients the ulcer had enlarged (up to 175%). Moreover, it might be expected that the outcomes would improve further if the follow-up was extended, given that the median surface area reduction from baseline was still increasing between week 8 and week 12. Together, the results support ABCB5^+^ MSCs as a promising candidate for adjunctive therapy of standard treatment-refractory DFUs and warrant further investigation to validate the observed benefit and to optime the dose regimen [68].

### 7.3. Recessive Dystrophic Epidermolysis Bullosa

Recessive Dystrophic Epidermolysis Bullosa (RDEB) is a rare, devastating, and life-threatening inherited disorder caused by lack of functional type VII collagen. The clinical presentation is characterized by an extremely weakened cutaneous mechanical stability, which manifests with painful and itchy non-healing or recurrent blistering wounds and various extracutaneous manifestations including esophageal and gastrointestinal mucosal scarring [182]. In consequence, patients suffer from a dramatically reduced quality of life and a high mortality risk from skin carcinoma [182,183]. A substantial contribution to the pathogenesis of severe inflammatory skin diseases including RDEB has been ascribed to sustained mechanical and/or oxidative stress-induced release of IL-1β by epidermal keratinocytes [184], which not only maintains skin inflammation but can spill over in the systemic circulation of RDEB patients to affect remote organs and promote life-threatening complications such as amyloidosis and kidney and heart involvement [184,185,186]. Thus, the capacity of ABCB5^+^ MSCs to dampen IL-1β-driven inflammation [11] (see Figure 4) makes them a promising candidate for disease-modifying treatment strategies in RDEB.

The generalized nature of RDEB, involving not only readily accessible skin wounds but also various internal, e.g., gastrointestinal, sites of basement membrane disruption, underlines the need for systemic treatment [187]. In this regard, intravenous administration of human ABCB5^+^ MSCs in a *Col7a1*^−/−^ NSG neonatal RDEB mouse model reduced RDEB pathology and markedly prolonged the animals’ lifespans via significant reduction in skin infiltration of pro-inflammatory M1 macrophages [102]. In human RDEB patients, intravenous infusions of allogeneic ABCB5^+^ MSCs distinctly increased wound closure parameters superior to historical placebo data. Importantly, about 75% of the closed wounds remained durably closed for at least 7 or even 9.5 weeks [188], which exceeds the typical natural recurrency period of RDEB wounds of roughly 3 weeks on average [189]. Even in the wounds that did not reach full closure during the 12-week efficacy follow-up period, intravenously infused ABCB5^+^ MSCs elicited significant mean decreases in wound size [188]. Moreover, the treatment significantly decelerated the formation of new wounds [188]. Additionally, ABCB5^+^ MSCs infusions significantly alleviated patient-perceived pruritus, which is consistently rated as the most bothersome symptom in RDEB, and reduced disease severity as measured by clinically meaningful reductions in two relevant disease severity scores, i.e., the Epidermolysis Bullosa Disease Activity and Scarring Index (EBDASI) activity score and the Instrument for Scoring Clinical Outcome of Research for Epidermolysis Bullosa (iscorEB) clinician score [166].

As RDEB is a progressive disorder, the observed improvements in disease activity, beyond alleviating current distressing symptoms, might also have contributed to the prevention of further accumulation of irreversible skin and organ damage. In line with this hypothesis, the EBDASI damage score, which captures accumulating damage, did not change with ABCB5^+^ MSC infusions during the 12-week follow-up period, indicating that no further damage accumulation had occurred [166]. Moreover, in view of the capacity of ABCB5^+^ MSCs to secrete type VII collagen (see Section 5.3.2. ECM Remodeling), one might speculate that long-term treatment with ABCB5^+^ MSCs might improve skin and mucosal structural integrity via accumulated deposition of type VII collagen [104]. In an upcoming pivotal phase III trial (NCT05464381), this hypothesis will be addressed by comparative collagen VII expression analysis in voluntary skin biopsies taken before treatment and during a 12-month follow-up period.

### 7.4. Further Possible Indications

While the current clinical development program for skin-derived ABCB5^+^ MSCs focusses on topical and systemic treatment of skin wounds and inflammatory dermatoses, the multifaceted modes of action open a range of potential further clinical indications for which urgent medical need exists.

In solid organ transplantation, the ability of ABCB5^+^ MSCs to inhibit alloantigen-dependent T-cell proliferation and induce of Treg expansion contributes to allograft protection and promotes a pro-tolerogenic environment, as was demonstrated in fully MHC-mismatched murine cardiac allotransplantation models, in which pretreatment with allogeneic ABCB5^+^ MSCs markedly prolonged allograft survival [10] (see Section 5.2.1. Effects on T-cells). In addition, making use of their anti-oxidative, anti-inflammatory, and proangiogenic capacities as well as their trophic efficacy on parenchymal cells, ABCB5^+^ MSCs might facilitate organ regeneration following ischemia-reperfusion injury in the setting of deceased organ donation and rejection reactions [190,191].

The potential of ABCB5^+^ MSCs to inhibit alloantigen-dependent T-cell proliferation and induce of Treg expansion could also be advantageous in the prevention and management of GvHD, a serious adverse reaction of allogeneic hematopoietic stem cell transplantation, which results from a systemic cytotoxic attack by alloantigen-specific donor T-cells on host tissues [192]. In addition to their effects on the adaptive immune system, ABCB5^+^ MSCs suppress, in inflamed tissues, IL-1β and its downstream effector TNF-α [11], two inflammasome components that are critical to GvHD development [193,194], while enhancing IL-10 [11], which is vital for GvHD limitation [195]. Furthermore, ABCB5^+^ MSCs can restrain ROS release from activated peripheral neutrophils [88] and might thus be able to alleviate ROS-mediated tissue damage by neutrophils recruited to the gastrointestinal tract during GvHD [196]. Finally, given that the pathogenesis of perpetuating organ and skin injury in GvHD has been attributed to endothelial vulnerability and damage associated with a strong decrease in VEGF levels during escalation of therapeutic immunosuppression [197,198,199,200,201], ABCB5^+^ MSCs might also attenuate GvHD via their strong pro-angiogenic potential [68,114].

Another potential indication relates to the ability of ABCB5^+^ MSCs to reverse metabolic reprogramming in polycystic kidney cells [132], which might qualify ABCB5^+^ MSCs as a potential candidate for improving kidney function in diseased kidney.

## 8. Challenges and Perspectives

Human skin-derived ABCB5^+^ MSCs, manufactured as a homogenous, standardized, highly pure ATMP with proven potency, bear potential to make a significant contribution toward fulfilling unmet needs of patients suffering from various inflammatory, immunological and/or degenerative diseases for which current treatments are lacking or for which currently available forms of therapy achieve unsatisfactory outcomes. Potential indications for ABCB5^+^ MSCs range from local inflammatory conditions such as chronic wounds to systemic, life-threatening diseases such as RDEB or GvHD.

The global prevalence of chronic wounds is expected to rise, fueled by an aging population and by increasing rates of risk factors such as obesity, cardiovascular disease, and diabetes. Consequently, effective advanced treatment approaches are urgently needed. At present, the efficacy and safety ABCB5^+^ MSCs for topical treatment of non-healing CVUs (see Section 7.1. Chronic Venous Ulcers) is being evaluated further in a randomized controlled phase IIb clinical trial with a dose-ranging design (NCT04971161), and a follow-up trial of ABCB5^+^ MSCs for topical treatment of non-healing DFUs (see Section 7.2. Diabetic Foot Ulcers) is in preparation. To facilitate early access for patients with treatment-refractory CVUs who are not eligible for enrolment in the ongoing phase IIb trial, the Paul Ehrlich Institute as the German competent authority has granted to ABCB5^+^ MSCs (AMESANAR^®^), as the second-ever somatic cell therapy medicinal product, national hospital exemption under Section 4b of the German Medicinal Products Act [202]. This regulatory approval enables that such patients can be treated with ABCB5^+^ MSCs even before the product has gained full market access through the elaborate and time-consuming centralized EU authorization pathway [203,204].

Clinical development of drugs for rare, “orphan” diseases such as RDEB is particularly challenging, owing to various factors that impede and limit clinical trials including small, heterogenous, and globally dispersed patient populations, limited knowledge of the natural history of disease, lack of clinically relevant endpoint definitions, and ethical issues (e.g., use of placebo), but also unfortunately due to investor-dependent commercial limitations given in the typically small target populations [205]. To stimulate development and marketing of ABCB5^+^ MSCs for the treatment of RDEB, both the US Food and Drug Administration and the European Medicines Agency have granted to ABCB5^+^ MSCs orphan medicinal product/drug designation, which offers several incentives including a 7-year (USA) or 10-year (EU) market exclusivity for RDEB [206,207]. In children, developing drugs for rare diseases is even more challenging, facing a particularly small and extremely heterogenous population ranging from preterm newborn infants to near adults [208]. Therefore, the successful negotiation of a pediatric investigation plan (PIP) is a prerequisite for application for marketing authorization of a new medicinal product in the EU. The approval of the PIP for ABCB5^+^ MSCs for RDEB by the Pediatric Committee of the European Medicines Agency granted in July 2021 represents a major milestone in this regard as it has now paved the way to develop a pivotal phase III trial (NCT05464381), which is expected to start in early 2023.

To allow for delivery of the cell numbers required for future clinical trials and subsequent potential market access, upscaling of cell production is essential. It is intended, by developing and validating a GMP-compliant expansion process for ABCB5^+^ MSCs run in a closed high-volume bioreactor system, to convert the cell production process from current labor-intensive monolayer to automated three-dimensional culture. Beyond delivering larger cell numbers, three-dimensional culture would also more closely resemble the physiological MSC environment than two-dimensional cultures, thereby potentially further enhancing the biological and therapeutic properties of the produced cells [209,210,211]. Moreover, specific priming strategies to sensitize and adapt the produced ABCB5^+^ MSCs to the hypoxic and/or inflammatory microenvironments that they encounter upon therapeutic administration, as well as additionally envisioned genetic bioengineering approaches to overexpress desired molecules such as type VII collagen lacking in RDEB, could help to further improve clinical efficacy, deliver more predictable outcomes, and unlock further new therapeutic indications.

## Figures and Tables

**Table 1:** MSC-based cell therapy products with marketing authorization in order of the date of (first) approval.

Trade Name Marketing Authorization Holder	Source Tissue	Indication, Route of Application	Country, Date of Approval
Cellgram-AMI Pharmicell	Bone marrow autologous	Acute myocardial infarction, intracoronary	Republic of Korea, Jul 2011
Cartistem Medipost	Umbilical cord blood allogeneic	Knee cartilage defects in patients with osteoarthritis, into the defect	Republic of Korea, Jan 2012
Cupistem Anterogen	Adipose tissue autologous	Crohn’s fistulas, intrafistular	Republic of Korea, Jan 2012
Remestemcel-L Mesoblast	Bone marrow allogeneic	Acute graft-versus-host disease, intravenous	New Zealand, Jun 2012 (approval lapsed) Canada, May 2015 (never marketed)
NeuroNata-R Corestem	Bone marrow, autologous	Amyotrophic lateral sclerosis, intrathecal	South Korea, Jul 2014
Temcell HS JCR Pharmaceuticals	Bone marrow allogeneic	Acute graft-versus-host disease, intravenous	Japan, Sep 2015
Stempeucel Stempeutics	Bone marrow, allogeneic	Critical limb ischemia due to thromboangiitis obliterans and peripheral artery disease, intramuscular	India, May 2016 (limited approval) Aug 2020 (full approval)
Alofisel Takeda	Adipose tissue, allogeneic	Complex perianal fistulas in patients with non-active or mildly active luminal Crohn’s disease, into the fistula tract tissue	EU, Mar 2018 Switzerland, Dec 2018 Israel Japan, Sep 2021
Stemirac Nipro	Bone marrow, autologous	Spinal cord injury, intravenous	Japan, Dec 2018 (conditional approval)

Data source: International Society for Cell and Gene Therapy (ISCT) [6].

**Table 3:** Treatment-related adverse events reported in clinical trials of ABCB5^+^ MSCs.

Clinical Trial	ABCB5^+^ MSC Doses		Treatment-Related Adverse Events				
NCT Identifier ^1^ Disease Follow-Up	Dosage	Total Number of Doses	Total Number of TRAEs	Event	Severity	Serious?	Outcome
NCT03257098 [152] CVU 12 months	1 × 10^6^ MSCs/cm^2^ topical	53	3	Increased wound exudation	Mild	No	Resolved without sequelae
				Erythema	Moderate	No	Resolved without sequelae
				Venous ulcer pain	Moderate	No	Resolved without sequelae
NCT03267784 [68] DFU 12 months	2 × 10^6^ MSCs/cm^2^ topical	39	0	n/a			n/a
NCT03529877 [166] RDEB 12 months	2 × 10^6^ MSCs/kg intravenous	46	3	Lymphadenopathy	Mild	No	Resolved without sequelae
				Hypersensitivity	Severe	Yes	Resolved without sequelae
				Hypersensitivity	Severe	Yes	Resolved without sequelae

^1^ See http://clinicaltrials.gov (accessed on 10 December 2022). CVU—chronic venous ulcer; DFU—diabetic foot ulcer; RDEB—recessive epidermolysis bullosa; TRAEs—treatment-related adverse events.

## Data Availability

Not applicable.
